# Draft Genome Sequence of *Rummeliibacillus* sp. Strain TYF005, a Physiologically Recalcitrant Bacterium with High Ethanol and Salt Tolerance Isolated from Spoilage Vinegar

**DOI:** 10.1128/MRA.00244-19

**Published:** 2019-08-01

**Authors:** Min Li, Yao Li, Xiaojun Fan, Yuhong Qin, Yongji He, Yongkang Lv

**Affiliations:** a College of Biomedical Engineering, Taiyuan University of Technology, Taiyuan, Shanxi, China; b Institute of Agricultural Products Processing, Shanxi Academy of Agricultural Sciences, Taiyuan, China; c Key Laboratory of Coal Science and Technology, Ministry of Education and Shanxi Province, Taiyuan University of Technology, Taiyuan, Shanxi, China; University of Maryland School of Medicine

## Abstract

Rummeliibacillus sp. strain TYF005 is a thermophilic bacterium with high ethanol (8% vol/vol) and salt (13% wt/vol) tolerance that was isolated from spoilage vinegar. Here, we report the draft genome sequence of this strain, which has 117 scaffolds with a total genome size of 3.7 Mb and a 34.4% GC content.

## ANNOUNCEMENT

The genus Rummeliibacillus was first described in the United States in 2009. R. stabekisii, a physiologically recalcitrant microorganism that came from the surface of a spacecraft, was the first species to be described (1). The genus comprises three species, namely, *R. stabekisii*, R. pycnus (1), and R. suwonensis (2). Although there have been a few studies on the genus, its application potential has been highlighted in biotechnology. For instance, it can convert palm oil mill effluent into terpolymer polyhydroxyalkanoate and biodiesel (3), and it has potential for biomineralization (4). Furthermore, a thermally stable arginase from *R. pycnus* is used in the production of l-ornithine (5, 6). In this study, we report the draft genome sequence of *Rummeliibacillus* sp. strain TYF005, which was isolated from spoilage vinegar in Shanxi, China, using de Man, Rogosa, and Sharpe (MRS) agar medium (7) with the dilution spread plate method (8).

**FIG 1:**
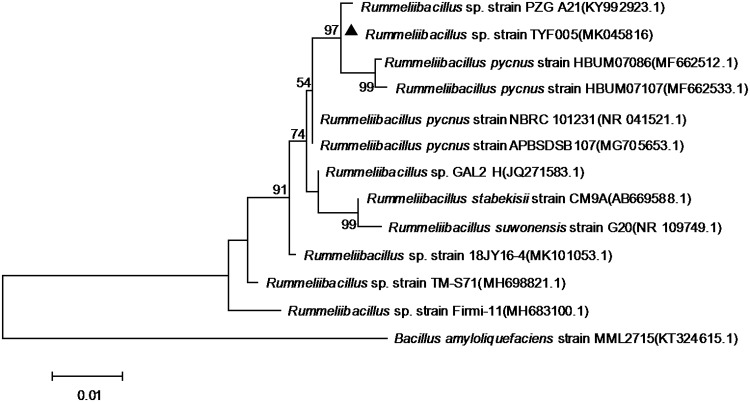
16S rRNA gene phylogeny of *Rummeliibacillus* sp. strain TYF005. The 16S rRNA gene sequences of related taxa were obtained from GenBank, and multiple alignments were performed with the CLUSTAL W program (22). Phylogenetic analysis based on the 16S rRNA sequence was conducted using the maximum likelihood method based on the Kimura 2-parameter model (23) in MEGA7 (24) with 1,000 replications in a bootstrap test. The initial tree for the heuristic search was obtained automatically by applying the neighbor-join and BioNJ algorithms to a matrix of pairwise distances estimated using the maximum composite likelihood (MCL) approach and then selecting the topology with the superior log likelihood value. Bootstrap values of >50% are shown. Bacillus amyloliquefaciens strain MML2715, which comes from the next closest genus, was used as an outgroup.

TFY005 was cultured in minimal medium (MM) broth (9) and MRS broth medium for 5 days with shaking at 200 rpm in an Erlenmeyer flask to investigate the ability to degrade corn straw and the tolerance to pH, temperature, alcohol, and NaCl (2). In all cases, the optical density at 600 nm (OD_600_) was measured to determine the cell growth. The strain TYF005 shows the ability to grow in MM culture medium containing 5% (wt/vol) natural corn straw powder as the sole carbon source. The optimal growth conditions in MRS broth include a pH of 5.0 to 8.5, alcohol and NaCl concentrations of 1 to 8% (vol/vol) and 1 to 13% (wt/vol), respectively, and a temperature of 30 to 55°C. The 16S rRNA gene was amplified by PCR with the universal primers 27F and 1492R as previously described (10). Based on analysis of a phylogenetic tree of the 16S rRNA for the most closely related species (Fig. 1), the strain was identified and designated *Rummeliibacillus* sp. strain TYF005.

A single colony of TYF005 was inoculated in MRS broth at 45°C for 24 hours, and 1.5 ml of the liquid culture was aliquoted for the DNA extraction and purification using a NucleoSpin tissue kit (TaKaRa Bio, Japan) following the manufacturer’s instructions. The extracted DNA was used with the TruSeq DNA sample prep kit (Illumina, CA, USA) to generate Illumina shotgun paired-end (400-bp) sequence libraries, which were sequenced on an Illumina HiSeq 2000 platform. A total of 7,627,629 raw paired-end reads with 2,303,543,958 bp were generated. Low-quality reads (quality score, 15), short reads (length, <25), and adaptors were removed, producing high-quality reads totaling 2,124,220,316 bp containing 6,884,863 paired-end reads and 714,099 single reads. The draft genome was assembled using SOAPdenovo v2.04 (11) (assembly parameter k = 31), and local hole filling and base correction for assembly results were acquired using GapCloser v1.12 (12), both with default settings. The protein sequences of the genes which were predicted using GLIMMER v3.02 (13) with default settings were searched against the nonredundant (NR) (14), Clusters of Orthologous Groups (COG) (15), STRING (16), gene ontology (GO) (17), and KEGG (18) databases using BLAST v2.2.28+ to obtain annotation information. rRNA genes and tRNA genes were predicted using Barrnap v0.4.2 and tRNAscan-SE v1.3.1 (19), both with default parameters. The sequencing protocol generated 170× coverage of the genome.

The genome of *Rummeliibacillus* sp. strain TYF005 has a size of 3.7 Mb with a GC content of 34.4%. The assembly resulted in 107 scaffolds (>10,00 bp), with the largest scaffold being 376,591 bp. The scaffold *N*_50_ and *N*_90_ values were 68,863 bp and 17,202 bp, respectively. The genome contains 3,610 genes, including 4 rRNA genes and 33 tRNA genes. The strain TYF005 has the ability to simultaneously utilize pentose and hexose. The genome contains genes for deferrochelatase (dye-decolorizing peroxidase) and laccase, which are involved in lignin degradation (20, 21). Furthermore, 253 genes are involved in biosynthesis of secondary metabolites. These results suggest that this strain has value in the conversion of straw-based biomass and various biotechnological processes, especially for some industrial processes requiring high temperature and high alcohol and salt concentrations.

### Data availability.

The whole-genome sequence (WGS) of *Rummeliibacillus* sp. strain TYF005 has been deposited under the BioProject number PRJNA421055 at DDBJ/ENA/GenBank and under the accession number QGPZ00000000. The version described in this paper is the first version. The raw sequencing reads are available as SRA data with the number SRS2763992.
